# The not quite Loudon–Fearn–Rarity–Tapster dip and its impact on the development of photonic quantum information

**DOI:** 10.1098/rsta.2024.0393

**Published:** 2024-12-24

**Authors:** John G. Rarity

**Affiliations:** ^1^ Department of Electrical and Electronic Engineering and H. H. Wills Physics Laboratory, QET Labs and Photonics and Quantum Group, University of Bristol, Bristol BS8 1UB, UK

**Keywords:** quantum interference, entanglement, pair photons

## Abstract

This paper presents a short history of the discovery by Rodney Loudon and Heidi Fearn of the counter-intuitive destructive interference effect occurring when two indistinguishable photons meet at a beamsplitter. This effect, commonly known as the Hong Ou Mandel effect, underpins much of present day photonic quantum information processing. Here I try to review its development from inception to present day proposals of million qubit photonic quantum computers.

This article is part of the theme issue ‘The quantum theory of Light’.

## 1. Introduction

This memorial paper begins with a short timeline of the discovery by Heidi Fearn and Rodney Loudon [1] of a counterintuitive destructive interference in the combining of two-photon probability amplitudes at a beamsplitter. When the beamsplitter has equal transmission and reflection probabilities (i.e. it is a 50:50 beamsplitter) and the two photons enter by two separate ports but are otherwise indistinguishable, then the probability of them exiting via separate ports drops to zero. Having seen this result early in 1987, Rodney slowly realized its significance over the course of the year and decided to publish after hearing of related theory results from the Martienson [2] and Mandel groups [3]. Then on a visit to the Rochester laboratory of Leonard Mandel in the summer of 1987, he guessed that an experiment might be in progress. When he met Paul Tapster and myself at the annual quantum electronics conference (QE8 in St Andrews, UK) in September of that year he convinced us of the significance of his result and we left to set up a quick experiment, getting our first results in early October. In those days, with no preprint archive, we kept up to date by post-lunch visits to the RSRE library browsing the latest journals. In late October, we found that the famous Hong Ou Mandel experiment was already reported in Physical Review Letters [4].

Only recently I met once again Zheyu (Jeff) Ou and he helped in getting the timeline right as to how and when they got their result. They were using a pair photon source producing only a few coincidence counts per second and they needed to overlap modes and arrival times at the beamsplitter in a similar way to making a classical white light interferometer. This proved a very difficult task. Each experiment took several hours, requiring the gathering of a significant number of coincidences at each position and scanning the region where suppression of coincidences (the ‘dip’) was expected to be. Eventually, after several unsuccessful weeks, Jeff found a hint of a dip in a noisy background (colloquially called ‘finding an arctic fox in the white out’). On further averaging this was confirmed as the dip. This was early summer 1987. It is my understanding that Heidi Fearn got the overlooked theory result in February 1987 and we saw Rodney in September, when he told us about the significance of Heidi’s result.

Although this relegated us to the second discoverers of the dip, delaying publication for a year [5], it set our group on a research direction which eventually led to the discovery of path entanglement [6], a key element of present day integrated photonics quantum computing [7,8].

In this paper, I present the development of the simple mathematics behind the HOM dip, the elements of which are expounded in the seminal Fearn and Loudon paper [1] and its extension to entanglement and a host of other two-photon interference effects [9–14]. In the mid 1990s, Rodney helped again in understanding that the photons did not need to have a shared history, inspiring us to interfere photons from different sources [15–18]. This of course led on to the extension of this destructive interference to perform entanglement of photons that have never met, leading to landmark experiments of quantum teleportation and entanglement swapping [19,20] and a host of further multiphoton interference experiments [21,22].

## 2. Some mathematical details

The discovery of the destructive interference at a beamsplitter showed the utility of the creation and annihilation operator [23] formalism for describing the interactions and interference of quantum states in classical apparatus. For the purposes of this paper, we introduce the creation operators $a_{i}^{+}$ for mode *i* which, when operating on a vacuum state, populates mode *i* with a single photon


(2.1) $$a_{i}^{+}\left|0\right\rangle ={\left|1\right\rangle }_{i}$$

Repeated operations with suitable normalization increase the photon number as in


(2.2) $$\frac{a_{i}^{+N}}{\sqrt{N!}}\left|\;0\right\rangle ={\left|\;N\right\rangle }_{i}.$$

In the opposite direction, annihilation operators $a_{i}$ reduce the photon number by one. The creation and annihilation operators have commutation relation


(2.3) $$\left[a_{i},a_{j}^{+}\right]=\delta \left(i,j\right).$$

**Figure 1.**
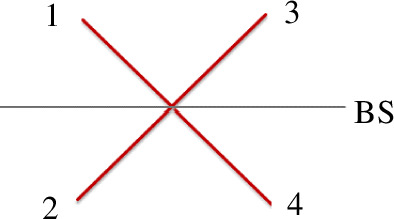
Input modes 1, 2 and output modes 3, 4 at a beamsplitter (BS).


As these operators are derived from the electric field operators used by Glauber [24] and others, this then allows them to evolve like fields propagate (within an optical mode) gaining phases and reflection/transmission amplitudes. We now consider the case of a beamsplitter, as illustrated in figure 1, specifically the case where two single indistinguishable photons enter ports 1 and 2. The input state can be represented as a product state of two 1-photon Fock states and using equation (2.1) this can then be reduced to creation operators acting on vacuum as shown in

(2.4) $$\left|\Psi_{in}\right\rangle ={\left|1\right\rangle }_{1}{\left|1\right\rangle }_{2}=a_{1}^{+}a_{2}^{+}\left|0\right\rangle .$$

Our beamsplitter is shown schematically in figure 1. Fearn and Loudon [1] noted that the field reflection ( ρ ) and transmission ( τ ) coefficients are constrained by energy conservation to satisfy


(2.5) $${\left|\tau \right|}^{2}+{\left|\rho \right|}^{2}=1\;\rho^{*}\tau +\rho \tau^{*}=0\;.$$

This definition assumes symmetry (unlike later Hadamard-based beamsplitter models) and is satisfied by choosing real amplitude coefficients t(=τ) and ir(=ρ) adding a π/2 phase shift on reflection to satisfy equation (2.5) [1]. On passing through the beamsplitter, the operators behave like fields as described above


(2.6) $$a_{1}^{+}\to ta_{4}^{+}+ira_{3}^{+}\text{ : }a_{2}^{+}\to ta_{3}^{+}+ira_{4}^{+}.$$

This then leads via simple steps to an output wavefunction


(2.7) $$\left|\Psi_{out}\right\rangle =\left(ta_{4}^{+}+ira_{3}^{+}\right)\left(ta_{3}^{+}+ira_{4}^{+}\right)\left|0\right\rangle =\left(t^{2}-r^{2}\right)|1\rangle_{3}|1\rangle_{4}+\sqrt{2}irt|2\rangle_{3}+\sqrt{2}irt|2\rangle_{4}.$$

The probability of seeing two-photon events in detectors placed after the beamsplitter


(2.8) $$\left\langle C\right\rangle =\left\langle \Psi \;\right|a_{k}^{+}a_{j}^{+}a_{j}a_{k}\left|\Psi \;\right\rangle \;\;\;k=4,\;j=3$$

then drops to zero when r=t . This was the key result of the Fearn and Loudon paper [1] with the first term in equation (2.7) showing a destructive interference effect between two photons that may never have interacted before. Fearn and Loudon’s table of outputs assuming symmetric outputs and real t,r is reproduced below (table 1).

**Table 1.** Reproduction of the table of photon output probabilities from a beamsplitter into paths 3, 4 given the various patterns of two-photon inputs first published in [1].

$|n_{1},n_{2}\rangle$	|2,0⟩	|1,1⟩	|0,2⟩
**P(2,0)**	$r^{4}$	$2r^{2}t^{2}$	$t^{4}$
**P(1,1)**	$2r^{2}t^{2}$	${\left(t^{2}-r^{2}\right)}^{2}$	$2r^{2}t^{2}$
**P(0,2)**	** $t^{4}$ **	** $2r^{2}t^{2}$ **	** $r^{4}$ **

Of course, so far we have only considered monochromatic photons but the Fearn and Loudon paper also provides the way in which we can define operators that populate spectro-temporal modes


(2.9) $$a^{+}\left(t\right)={\left(2\pi \right)}^{-1/2}\int_{-\infty }^{\infty }a^{+}\left(\omega \right)e^{i\omega t}d\omega .$$

Again satisfying the commutation rule


(2.10)) $$\left[a\left(\omega \right),a^{+}\left(\omega^{\prime }\right)\right]=\delta \left(\omega ,\omega^{\prime }\right).$$

Spectrally limited photons can then be defined by a normalized spectral function α(ω)


(2.11) $$\begin{array}{l} a_{\alpha }^{+}=\int_{-\infty }^{\infty }\alpha \left(\omega \right)a^{+}\left(\omega \right)d\omega ,\\ \;\;\int_{-\infty }^{\infty }|\alpha \left(\omega \right){|}^{2}d\omega =1. \end{array}$$

For realistic detectors with temporal resolution ΔT the probability of a coincidence detection is then calculated from


(2.12) $$C_{12}\left(\Delta T\right)\propto \iint_{-\Delta T/2}^{\Delta T/2}\left\langle \Psi \right|a_{i}^{+}\left(0\right)a_{j}^{+}\left(\tau \right)a_{j}\left(0\right)a_{i}\left(\tau \right)\left|\Psi \right\rangle \text{d}\tau_{i}\text{d}\tau_{j}.$$

Now this formula provides the means to calculate the temporal shape of a dip in coincidences at the point when finite length photons overlap at a beamsplitter. All of these equations are present in the seminal Fearn and Loudon paper [1] and most experimental work in quantum interference experiments since then is based on their application. I attempt to illustrate this with examples in the following.

## 3. Early experiments

**Figure 2.**
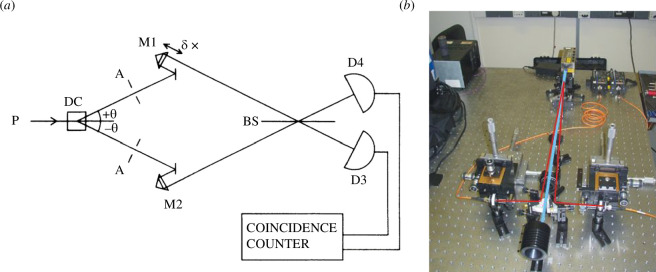
(*a*) Schematic of our first HOM dip experiments reproduced from [25]. Pump photons (P) pass through a nonlinear crystal (DC) phasematched to create degenerate photon pairs at angles ±θ from the pump. The emitted beam divergence is confined by apertures A which define the photon spectra. The light is then redirected towards a 50:50 beamsplitter BS by mirror and retroflector units M1 and M2. M1 has adjustable path delay *δ*x allowing us to scan the dip. After the beamsplitter photons are detected in photon counting detector D3 and D4 and electronic photodetection pulses are directed to a coincidence counter. (*b*) Picture of a more recent parametric pair photon source in the laboratory. Pairs are created in a nonlinear crystal (beta barium borate BBO) pumped by a violet laser beam (405 nm). In this arrangement, the pairs are reflected into lenses focused onto single mode fibres all mounted onto three-dimensional alignment stages to select the degenerate photon pairs. Effectively the projected mode of the fibre replaces the apertures shown in (*a*).

In the first experiments, the source of coincident photon pairs was spontaneous parametric downconversion where photons from a bright coherent pump source (laser) have a small probability of being downconverted to pairs of photons in a nonlinear crystal. Our version of the experiment is illustrated in figure 2*a*. The process is energy conserving, the sum of downconverted photon energies is equal to the energy of the pump photon. Although the sum of the pair photon energies are equal to a well-defined energy, the phase matching process in the nonlinear crystals used is usually much broader band. This leads to photon-pair spectra that are centred around the pump energy in an anticorrelated way. A higher energy photon is always partnered with a lower energy photon and vice versa. It also means the underlying generated state is entangled in energy.

However the first experiments missed the subtleties of this energy entanglement, focusing instead on the fact that the relatively wide band photons led to very short temporal dips inaccessible to standard photon counting detectors, a fact reflected in the title of the HOM dip paper [4]. In pursuing our target of demonstrating in the laboratory the shortest temporal dip, we reached a dip full width half maximum of 39 fs corresponding to a pathlength difference change of 11.7 µm [5]. In trying to work out why the dip width could not be any shorter, we discovered that the frequency anticorrelation inherent in the energy matching cancelled out all first-order dispersion effects. The significance of this result was pointed out to us by later authors [9]. The minimum dip width measured was likely due to higher order dispersion effects, notably group velocity dispersion which played a significant role in later short pulse pump experiments.

**Figure 3.**
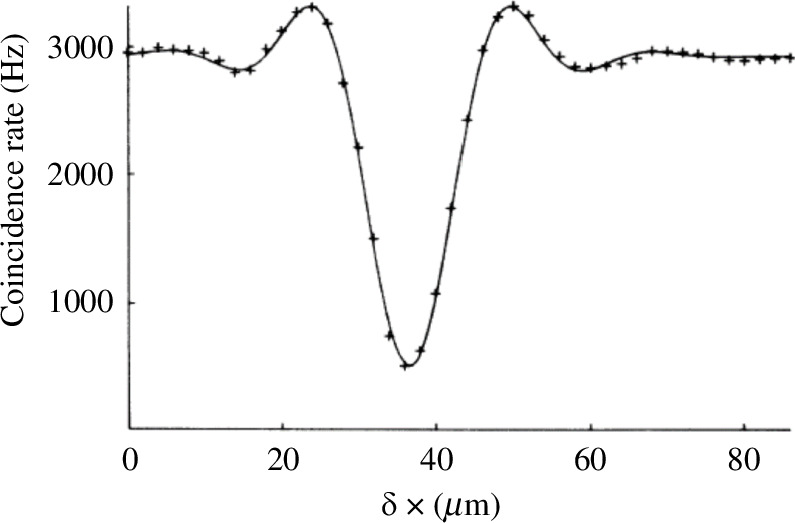
Sinc-shaped coincidence dip arising when apertures in figure 2*a* define a ‘top hat’ joint spectrum of the photon pair. Reproduced from [25].

To create very short temporal dips, we were using slits of increasing width to increase the photon bandwidth. The resulting ‘top hat’ spectrum led to us seeing sinc-like interference curves as seen in figure 3 where the coincidence rate increased above the expected rate for uncorrelated photons. This highlighted the fact that the shape of the temporal dip is governed by the Fourier transform of the photons’ spectra.

**Figure 4.**
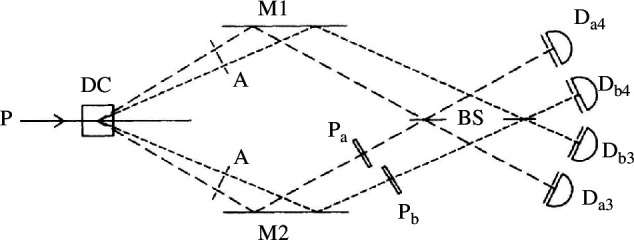
Two colour pair photon interference apparatus [6] is identical to figure 2*a* apart from now the aperture consists of two slits creating two-colour photons labelled a and b. Separating out the colours into four detection channels D_a4_, , D_a3_, D_b3_, D_b4_ allows path beating between colours and at zero path difference phase plates P_a_ and P_b_ can be used in an analagous way to rotating polarizers allowing the demonstration of Bell inequality violation.

**Figure 5.**
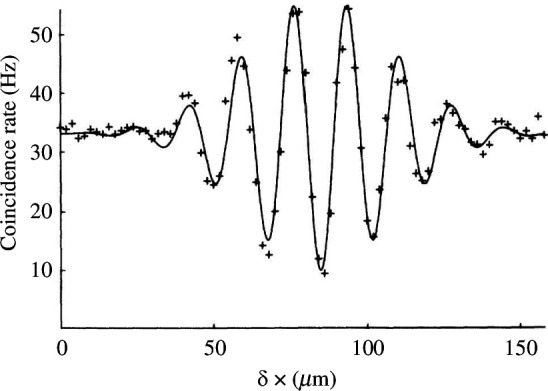
Typical coincidence count as a function of path length difference when measuring between D_a3_ and D_b4._. Similar curves were obtained from a4, b3 coincidences while a3, b3 produces an identical oscillation π out of phase. Adjusting the phase plates P_a_ and P_b_ changes the phase of this curve moving the central point from a minimum to a maximum. Focusing on the central point and measuring all coincidence patterns at suitable chosen phases, a demonstration of Bell inequality violations was achieved in [6].

This led to the idea of creating two-colour photon pairs by placing a central block in our apertures creating two slits as shown in figure 4. Now the destructive interference ‘dip’ shows a beating effect in the coincidence rate as a function of path length difference as shown in figure 5. We can also recast this experiment by realizing that the result is retained when we separate the detectors into a long wavelength photon detection channel (a) and a short wavelength interference channel (b). Now we see that after recombination at the beamsplitter we are interfering two possible ways of creating our non-degenerate a–b photon pair. The phase of the interference function shown in figure 5 can thus be shifted by adding waveplates P_a_ and P_b_ in either channel, allowing the central (zero pathlength difference dip) to be adjusted to be a peak in coincidences. It also becomes clear that the a and b photons are now spatially separable and could be detected separately in remote locations. Now we are demonstrating the interference of two indistinguishable pairs of paths. Referring to figure 4, we are seeing interference between the pair amplitude travelling by 2a−1b and the amplitude travelling by 1a−2b represented by the state.

**Figure 6.**
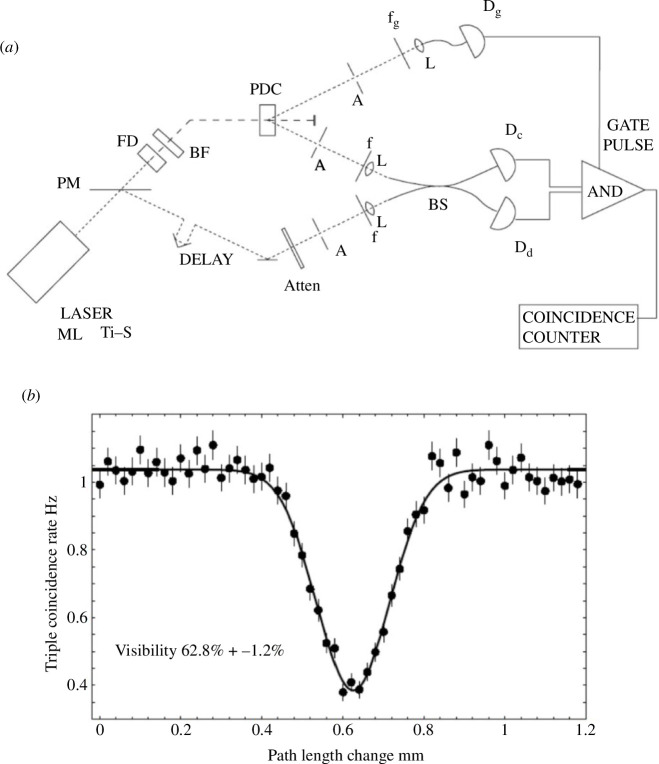
Interference of separate sources (*a*) experimental arrangement from [18]. A mode-locked Ti-Sapphire laser ML-Ts producing approximately 100 fs pulses at 100 MHz repetition rate centred at 815 nm wavelength passes through a pick-off mirror and is frequency double (FD) to 407.5 nm with the fundamental blocked (BF). The beam pumps a BBO parametric downconversion crystal (PDC) creating degenerate pairs at symmetric approximately 3° angles to the pump. One of the pair passes to a gate photon counting detector D_g_ via aperture A, bandpass filter f_g_ and lensed fibre while the other passes to one input of a fibre beamsplitter via aperture A, filter f and lense L. The pick-off mirror meanwhile directs a small fraction of the original pump beam via a variable delay and identical filter f to the other input to the fibre beamsplitter. Detections in D_g_ are then used to herald single photons entering the beamsplitter and subsequently measuring a coincidence behind the beamsplitter in D_c_ and D_d_ implies a single photon and weak coherent pulse entered the beamsplitter. (*b*) On measuring the rate of triple coincidences as a function of delay, we see a triple coincidence rate of roughly 1 per second until the delays match. Around this point, we see a destructive interference dip showing >60% visibility confirming the non-classical nature of the interference.


(3.1) $${\left|\Psi \right\rangle }_{in}=\frac{1}{\sqrt{2}}\left(e^{i\emptyset_{b}}{\left|1\right\rangle }_{a1}{\left|1\right\rangle }_{b2}+e^{i\emptyset_{a}}{\left|1\right\rangle }_{a2\;}{\left|1\right\rangle }_{b1}\right).$$

The normalized coincidence rates measured at detectors sitting behind the beamsplitter at nominal zero path difference are then expected to be


(3.2) $$C_{ab}\left(0\right)\propto \;1\mp Vcos\left(\emptyset_{a}-\emptyset_{b}\right),$$

where *V* is a fringe visibility dependent on beamsplitter being 50:50 and the mode overlap at the beamsplitter. $\emptyset_{a},\emptyset_{b}$ are phases imparted by phase plates P_a_ and P_b_. The minus sign corresponds to detections in outputs 3a, 4b (or 3b, 4a) of the beamsplitter while the plus sign describes 4a, 4b or 3a, 3b coincidences. The variations of coincidence rates with phase in equation (3.2) are mathematically equivalent to the effect of rotation of polarizers in previous polarization-based Bell inequalities [26]. Hence this two-path interference can be mapped onto a test for local realistic phases between amplitudes in arms a1–a2 and b1–b2, after photon emission, leading to the first demonstration of a path (momentum) entanglement Bell inequality [7].

The study of pair photon interference phenomena then expanded rapidly with key advances such as dispersion cancellation [6], post-selected polarization entanglement [10], pair photon interference in Mach Zehnder interferometers [11], Bell violations in separated unbalanced interferometers [12], induced coherence [13] and frustrated pair photon spontaneous emission [14]. Pair photon interference has remained a productive research area up until the present day and I am always surprised to see new phenomena discovered every few years. However after initial experiments we became interested in looking beyond two photons to experiments involving three or more photons and generalizations of entanglement beyond pairs. This was further emphasized in discussions with Rodney who reminded us that the theory in their original paper did not require the interfering photons to have any shared history. This led us to develop ways of interfering photons generated in separate sources [15,16]. It became clear that heralded single photons from CW sources gated by electronics with timing resolution on the order of a nanosecond would contain many thousands of coherence lengths even from nanometre bandwidth filtered sources washing out any interference. To ensure high visibility interference it is necessary to optically gate the photon pairs using a short pulse (100 fs or so) laser pumping the crystal. Also, the generation rate of pairs of heralded photons was limited and thus the chance of overlap was low, leading to extremely low coincidence rates in potential experiments (approx. 1 per second). We already knew that the attenuated laser beam could be an approximate source of single photons and with Rodney we showed that two-photon interference between a single photon and a weak coherent state could lead to high visibility interference. Replacing the pump with a frequency doubled femtosecond Ti-sapphire laser then allowed us to demonstrate the ‘Fearn Loudon’ dip existed even when the sources were different, albeit in this case we used the attenuated undoubled pump as our weak coherent state. The experiment is illustrated in figure 6*a* with our key result showing a dip visibility greater that 50%, proving its non-classical nature, shown in figure 6*b*. The experiment was slowed by a poorly performing pump laser but eventually we began to report first results in 1996 conferences [17] and an arXiv co-authored with Rodney in 1997, which for various reasons was formally published some years later in 2005 [18].

The combination of this demonstration and the discovery that destructive interference at the beamsplitter could be configured to perform a post-selected Bell measurement [27] then set the scene for the seminal experiments demonstrating teleportation [19] and entanglement swapping [20]. This led on to a large subfield demonstrating various multiphoton interference effects reviewed in [21] and in a book authored by Jeff Ou [22]. However, the teleportation and entanglement swapping papers were also the first application of post-selected two-photon interference used to develop quantum information processing primitives and I describe developments in that area in the next section.

## 4. Application to quantum computing

When we change the values of r and t in equation (2.7) we move away from total destructive interference, creating a reduced probability for two photons to exit the beamsplitter in coincidence. One special situation occurs when


(4.1) $$t=\frac{1}{\sqrt{3}}\;\;r=\frac{2}{\sqrt{3}}.$$

**Figure 7.**
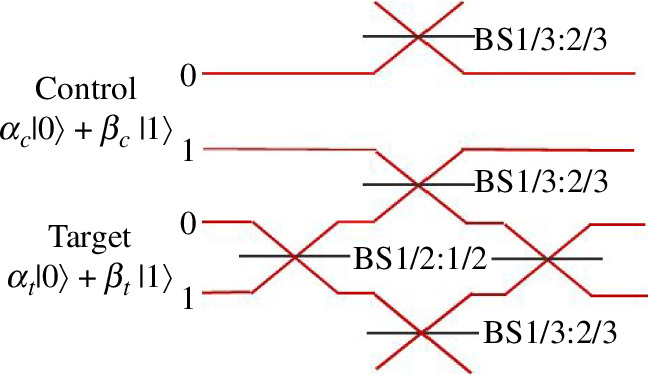
Schematic of the post-selected CNOT gate. Arbitrary target and control qubits are path encoded. The target photon is input into a balanced interferometer (placed between two Hadamard gates formed by 50:50 beamsplitters). Inside the interferometer, the target qubit in one arm is mixed with the control qubit line 1 in a 1/3:2/3 beamsplitter. This imparts a conditional phase shift flipping the target photon output lines whenever control qubit is a ‘1’. The partial destructive interference means that this only occurs 1/9th of the time and only when we post-select on coincident detections between control and target outputs. To balance the losses due to this destructive interference in the central beamsplitter loss is added by including extra 1/3:2/3 beamsplitters (configured to reflect 1/3 intensity here) in the ‘0’ control mode and the lower target mode of the interferometer.

In intensity terms this is a 1/3:2/3 beamsplitter. For this case, when we post-select only those results where coincident detections are seen between outputs 3 and 4, equation (2.7) becomes


(4.2) $$\left|\Psi_{out}\right\rangle =-\frac{1}{\sqrt{9}}{\left|1\right\rangle }_{3}{\left|1\right\rangle }_{4}$$

and on noting that −1= $e^{i\pi }$ this corresponding to a phase shift of π applied to the state. This post-selected conditional phase shift occurs with probability 1/9^th^ (11%) and forms the core of the first experimentally realizable quantum CNOT gate. The scheme is illustrated in figure 7 where a control and target photon are encoded in paths 0, 1 to create two qubits in a product state. The target photon is input into a Mach Zehnder interferometer suitably balanced to output an undisturbed state in the case when no photon is on control line ‘1’. When there is a photon on control line ‘1’ then 1/9^th^ of the time two photons exit the 1/3:2/3 beamsplitter carrying a conditional phase. This phase then flips the outputs from the target interferometer performing the CNOT operations. The additional 1/3:2/3 beamsplitters serve to reduce the amplitude of the non-interacting amplitudes to maintain the fidelity of the gate theoretically at unity, with 11% efficiency. This gate is a simplified version of the Knill, Laflamme and Milburn conditional CNOT gate first introduced in 2001 [28].

**Figure 8.**
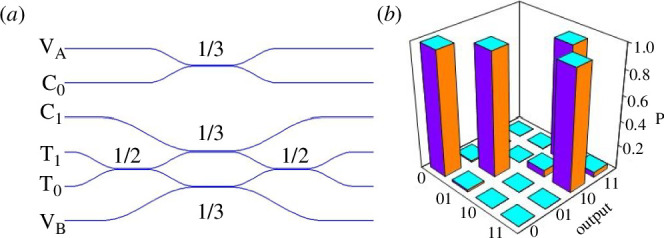
(*a*) Schematic of a waveguide realization of a post-selected CNOT gate. Waveguide couplers replace the beamsplitters of figure 7 with reflectivities as shown. Input C_0/1_ are the control and T_0/1_ the target qubit modes with vacuum ports V_AB_ used to check the reflectivities and stability of the interferometer (*b*) Measured fidelity of the waveguide CNOT gate. In this representation, an identity gate would produce diagonal unit height matrix components. The very slight imperfections of this gate appear as very small residual matrix elements on the diagonal with near unit height of the switched 10–11 and 11–10 values. Figure reproduced from [7].

In a more formal presentation of gate operation, we find that the general product state input


(4.3) $$|\Psi \;\rangle_{in}=\left(\alpha |0\rangle_{t}+\beta |1\rangle_{t}\right)\left(\alpha_{c}|0\rangle_{c}+\beta |1\rangle_{c}\right)$$

with amplitudes for target α,β and control $\alpha_{c},\beta_{c}$ qubits is transformed to the general entangled state representing the output of a CNOT


(4.4) $$|\Psi \rangle_{out}=\frac{1}{3}(\alpha \alpha_{c}|0\rangle_{t}|0\rangle_{c}+\alpha \beta_{c}|1\rangle_{t}|1\rangle_{c}+\beta \alpha_{c}|1\rangle_{t}|0\rangle_{c}+\beta \beta_{c}|0\rangle_{t}|1\rangle_{c}$$

when we post-select on coincident detections. This was first demonstrated in bulk optics [29] requiring a subtle common path interferometer arrangement to guarantee stability in the target interferometer. Then the simple idea of moving to an integrated photonics version of the gate was introduced by Jeremy O’Brien and his student Alberto Politi. They designed a silica-based photonic CNOT chip represented schematically in figure 8*a*, then used a bulk parametric source (see figure 2*b*) to generate photon pairs that were efficiently coupled into the control and target waveguides leading to demonstration of high fidelity post-selected CNOT gate operation [7]. The high fidelity CNOT operation was possible due to the high interferometric stability and naturally single mode properties of waveguides and signalled the future for quantum photonic logic. The high fidelity of the gate in the computational basis is shown in figure 8*b*.

**Figure 9.**
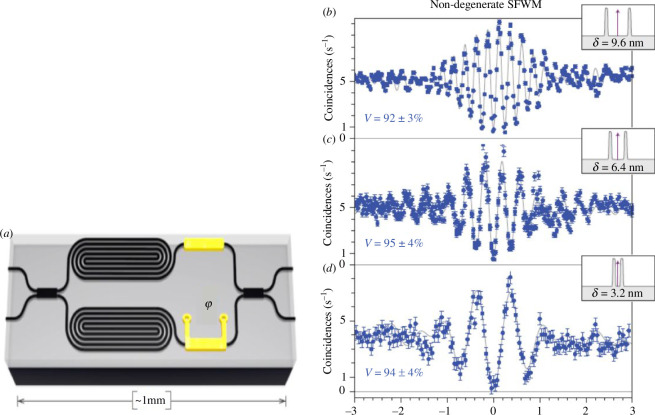
(*a*) Silicon chip based pair photon sources created by long waveguide coils where broad band non-degenerate pair photon generation happens either side of the narrow band pulsed pump beam (1550 nm). Here the pairs are generated in two coherently pumped sources and recombined at a beamsplitter with variable phase leading to a variety of two-photon interference effects. Shown here in (*b, c, d*) are measurements of two-colour beating as a function of path length difference seen in an off chip interferometer. By using DWDM filters to select pairs at a range of wavelength separations, we then can see changes in the beat frequency of the coincidences versus pathlength difference in a analogous way to those seen in the earlier two-colour photon beating experiments in figure 5 and [6]. Figures reproduced from [8].

It was quickly realized that despite the silica chip waveguides being very low loss, their structure and index contrast are very similar to optical fibres and this restricts circuits to have very large radius bends leading to centimetre scale circuits for single gates. This led to a quick evolution to silicon photonics where foundry processes from micro-electronics are highly developed and non-quantum photonic circuits are being developed at a high pace. Silicon also has a high third-order nonlinearity and it is possible to achieve phase matching for four wave mixing based parametric sources. Non-degenerate pairs of photons are created around a 1.55 µm pump laser with two photons annihilated from the pump for every pair created. This makes the process quadratic in pump power but this requirement for high intensities is achieved from the strong waveguide confinement and pulsing the pump [30]. The pair-photons are created with wavelengths above and below the pump wavelength satisfying energy matching conditions. These non-degenerate photons cannot be used to demonstrate interference but in analogy to the path entanglement experiment of figure 4 we can easily make two coherently pumped sources that overlap the degenerate photons at separate beamsplitters to create path entanglement. The first experiments of this type were performed by Silverstone *et al.* [8], where both long and short wavelength photons were interfered on a single beamsplitter as shown in figure 9. Due to fabrication limitations at the time (low loss crossovers were not yet developed), interference at separate beamsplitters was not possible but a range of HOM and two-photon interference effects were demonstrated with source now on chip and beating experiments were demonstrated in an off-chip interferometer (figure 9*b*–*d*).

**Figure 10.**
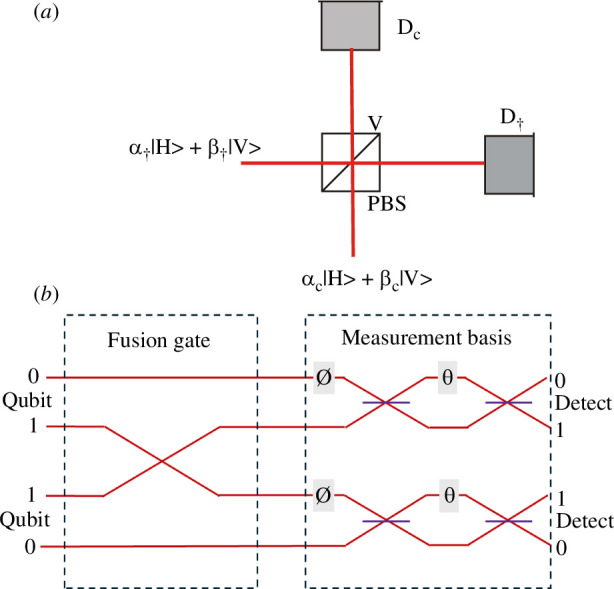
(*a*) Principle of the polarization-based fusion gate. Photons encoding arbitrary target and control qubits enter the polarizing beamsplitter that reflects V-polarized photons and transmits H-polarized photons. When identical photons encoding arbitrary polarization encoded qubits are input to the gate, the post-selected photons travelling in the output target and control lines to polarization measuring detectors D_c_ , D_t_ are entangled. (*b*) Schematic of a path implementation suitable for an integrated quantum photonics experiment. This is effected simply by swapping the 1 value waveguides at a low loss, low crosstalk waveguide crossover. This highlights that interference does not happen in the fusion gate but occurs when we superpose 1 and 0 lines in a variable Mach Zehnder to perform arbitrary measurements as shown. Phases ∅,θ define an arbitrary polar coordinate axis of the Bloch sphere. For instance a Pauli *Z* measurement requires: ∅=0,θ=0 , Pauli *X*: ∅=0,θ=π/2 , and Pauli *Y*: ∅=π/2,θ=π/2 .


A further exploitation of the interference between indistinguishable photons has been the development of various forms of the parity gate [31], later named the fusion gate [32]. The post-selected polarization version entangles product state photons in a polarizing beamsplitter as shown in figure 10*a*. Essentially the input product state is entangled when we post-select on coincident detection in control and target arms;

(4.5) $$\begin{array}{l} |\Psi \rangle_{in}=\left(\alpha_{t}|H\rangle_{t}+\beta_{t}|V\rangle_{t}\right)\left(\alpha_{c}|H\rangle_{c}+\beta_{c}|V\rangle_{c}\right)\to \\ \;\;\;|\Psi \rangle_{out}=\left(\alpha_{t}\alpha_{c}|H\rangle_{t}|H\rangle_{c}+\beta_{t}\beta_{c}|V\rangle_{t}|V\rangle_{c}\right). \end{array}$$

Also shown in figure 10*b* is a path encoded version suitable for an integrated photonics realization of the fusion gate which at first sight only requires a swap of one pair of lines (here the Qubit 1 lines) and does not apparently involve interference. However interference occurs when measuring in any basis other than the computational Pauli Z basis. This is shown by adding a suitable interferometer and phases on the right-hand side of figure 10*b*.

**Figure 11.**
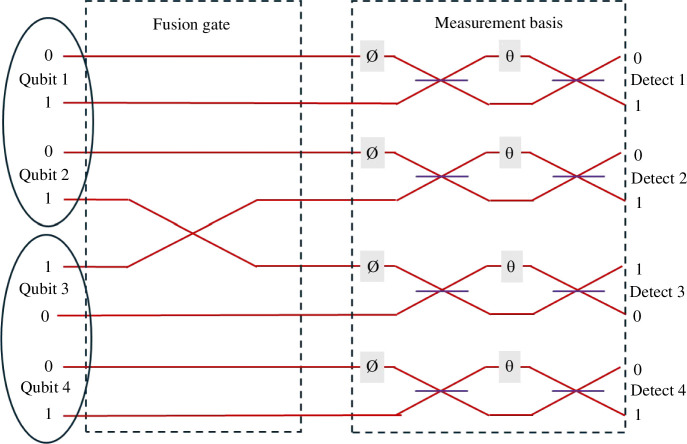
Schematic of the layout of the four-photon entanglement using path encoding and the fusion gate introduced in figure 10*b*. Two entangled photon pairs (1, 2 and 3, 4) are generated using a circuit similar to that shown in figure 9. Qubits 2 and 3 are directed through the fusion gate where their ‘1’ lines are switched in a low loss crossover. The post-selected fourfold coincidences in outputs Detect 1, 2, 3, 4 are then measured to be in a GHZ state. One can also view the measurement basis circuit as a set of local operations converting the basic GHZ state into a variety of cluster states as described in [33].

The fusion gate is useful as it allows us to build up higher order entanglement through the fusion of two entangled states to create four-photon entangled states. Taking the product of two pairwise entangled states,


(4.6) $$\begin{array}{l} |\Psi \rangle_{in}=\frac{1}{2}\left(|0\rangle_{1}|0\rangle_{2}+|1\rangle_{1}|1\rangle_{2}\right)\times \left(|0\rangle_{3}|0\rangle_{4}+|1\rangle_{3}|1\rangle_{4}\right)\to \\ \;\;\;\;\;\;|\Psi \rangle_{out}=\frac{1}{2}\left(|0\rangle_{1}|0\rangle_{2}|0\rangle_{3}|0\rangle_{4}+|1\rangle_{1}|1\rangle_{2}|1\rangle_{3}|1\rangle_{4}\right), \end{array}$$

the post-selected fourfold coincidence state is a fourfold GHZ state when we combine (fuse) photons 2 and 3 in the simple fusion gate illustrated in figure 10*b*. A schematic of the full fourfold fusion scheme is shown in figure 11. An experiment demonstration of four photon entanglements on chip was realized in 2019 [33]. The experiment showed the ease of adjusting the state via local operations identical to those used for selecting measurement basis to create various four-photon graph states.

This has led on to more and more complex entanglement arrangements including teleportation on-chip and between chips [34] and higher dimensional eight-qubit entanglement [35]. The state of the integrated quantum photonics field was reviewed in 2020 [36] covering the early development of cluster state computing primitives enabled by the fusion gates described above. This work is motivated by the initial idea for cluster state quantum computation introduced by Rausendorf and Breigel [37] that signposts the future for on-chip quantum information processing and scalable architectures for large-scale error corrected quantum computation [38,39]. Here the engineering challenges are of creating ultra-low loss circuits, efficient deterministic single/multi-photon sources, near unit fidelity gates and on chip unit efficiency detectors. In our recent work, we have focused on the improvement of the fidelity of quantum interference which is key to all of the required gates. In the move from bulk to integrated photon circuits the dramatic increase in fidelity arose primarily from the fact that pairs were created in a single spatial mode that could be overlapped with near identical spatial modes in waveguide couplers. However, when interfering pulsed pair sources to create higher photon number entangled states the fidelity of the interference is also dependent on the heralded photons being created in a single spectro-temporal mode. In this case, the joint spectral amplitude of the pair photon state becomes factorable, and thus uncorrelated. More importantly, no information about the heralded photon is gained by detection of the heralding photon. The technique of Schmidt decomposition can be used to estimate the number of spectro-temporal modes by decomposing the measured or predicted joint spectral amplitudes into a weighted sum of orthogonal product modes. The deviation from single moded can then be estimated and the maximum interference visibility predicted. This was first discussed by Grice *et al.* [40] applied to crystal-based parametric downconversion. The subtleties of designing these single moded product state pair photon sources are related to the phase matching conditions in the crystal or waveguide source. High purity spectrally uncorrelated sources of pair photons have been developed using modal phase matching [41] and by engineering a ‘soft edge’ nonlinear coupling length using two colour pump pulses travelling at different group velocities. Our recent work has engineered a source using coupled ring resonators to build a ‘photonic molecule’ pair photon source. This allows high brightness pair photon generation with high heralding efficiency (>94%) while retaining highly uncorrelated photon spectra (>99% purity) ideal for making high fidelity fusion gates [42].

However to create a fully integrated scalable platform suitable for large-scale quantum computation will require further engineering to improve fidelities, heralding efficiencies, detection efficiencies etcetera beyond 99%. Even with the ultimate fidelity, the lowest losses and highest detector efficiencies the resulting error rates in fundamental operations will require many photons to encode error protected qubits [38,39]. The maximum efficiency of post-selected gates of 75% then also requires a huge overhead and eventually a dynamic percolation model cluster state computation with rapid electronic feed forward of measurement results. The requirement for millions of photonic qubits and complex high-speed control electronic circuitry all at or below 4K presents significant engineering challenges including improving the fabrication tolerances of photonic integrated circuits to provide repeatability, low loss high-speed switching and integration with complex high-speed electronic control circuitry. This has led to the work moving largely to venture capital funded companies, in particular PsiQuantum formed by elements of the Thompson and O’Brien teams from Bristol and the Rudolph group from Imperial. The company has raised over a billion dollars now from venture capital, and governments (UK and Australia) and using bespoke silicon and SiN fabrication facilities have made great strides to realizing the individual element performance to make a scalable quantum optical computer [39]. Initially working in ‘stealth’ mode the company has recently revealed striking progress towards developing a low loss platform capable of scaling to the required complexity [43].

## 5. Conclusion

The first conclusion was that the work of Fearn and Loudon [1] requires the rewriting of Dirac’s statements: ‘*Each photon then interferes only with itself. Interference between different photons never occurs*,’ as quoted from the 1958 edition of his book [44]. This then became the ‘scripture’ describing the early single quantum interference experiments. The fact that separate radio frequency sources were always known to classically interfere was overlooked by the quantum community. The work of Pfleegor and Mandel, interfering separate weak laser beams [45], reconfirmed that classical separate sources of electromagnetic waves do interfere even at multi-terahertz frequencies. Then, of course, the ensuing pair photon interference experiments [4–6,9–14] confirmed this statement needed a careful updating to include multiple photon and multiple path interferences. Glauber wrote a very nice comment on how to interpret interference in the modern quantum world in [46] which I quote here:

*The things which interfere in quantum mechanics are not particles. They are probability amplitudes for certain events. It is the fact that probability amplitudes add up like complex numbers that is responsible for all quantum mechanical interferences.*

Bearing this in mind, we realize that the quantum mechanical speed up inherent in the various forms of quantum computation is the result of just this adding up of probability amplitudes, in some cases in their thousands/millions. The challenge is then only in the engineering of the high precision circuits (photonic and electronic) that allow the constructive interferences to build, pointing to the solution of the problem posed. For instance, in the case of Schor’s algorithm for factorizing large products of primes [47], this is the period of a function which then constrains the search space to one solvable by a polynomial function. It is the clear sighted fundamental expositions of the simplest of these interferences by Rodney Loudon [1] and his contemporaries [2,3] that underpins all of this extraordinary progress.

## Data Availability

This article has no additional data.

## References

[1] Fearn H , Loudon R . 1987 Quantum theory of the lossless beam splitter. Opt. Commun. **64** , 485–490. (10.1016/0030-4018(87)90275-6)

[2] Prasad S , Scully MO , Martienssen W . 1987 A quantum description of the beam splitter. Opt. Commun. **62** , 139–145. (10.1016/0030-4018(87)90015-0)

[3] Ou ZY , Hong CK , Mandel L . 1987 Relation between input and output states for a beam splitter. Opt. Commun. **63** , 118–122. (10.1016/0030-4018(87)90271-9)

[4] Hong CK , Ou ZY , Mandel L . 1987 Measurement of subpicosecond time intervals between two photons by interference. Phys. Rev. Lett. **59** , 2044–2046. (10.1103/PhysRevLett.59.2044) 10035403

[5] Rarity JG , Tapster PR . 1988 Non-classical effects in parametric downconversion. In Photons and fluctuations (eds ER Pike , H Walther ), p. 122. London: Taylor and Francis. (10.1201/9781003069539)

[6] Rarity JG , Tapster PR . 1990 Experimental violation of bell’s inequality based on phase and momentum. Phys. Rev. Lett. **64** , 2495–2498. (10.1103/PhysRevLett.64.2495) 10041727

[7] Politi A , Cryan MJ , Rarity JG , Yu S , O’Brien JL . 2008 Silica-on-silicon waveguide quantum circuits. Science **320** , 646–649. (10.1126/science.1155441) 18369104

[8] Silverstone J *et al* . 2014 On-chip quantum interference between two silicon waveguide sources. Nat. Photonics. **8** , 104. (10.1038/nphoton.2013.339)

[9] Franson JD . 1992 Nonlocal cancellation of dispersion. Phys. Rev. A **45** , 3126–3132. (10.1103/physreva.45.3126) 9907348

[10] Shih YH , Alley CO . 1988 New Type of Einstein-Podolsky-Rosen-Bohm Experiment Using Pairs of Light Quanta Produced by Optical Parametric Down Conversion. Phys. Rev. Lett. **61** , 2921. (10.1103/PhysRevLett.61.2921) 10039265

[11] Rarity JG , Tapster PR , Jakeman E , Larchuk T , Campos RA , Teich MC , Saleh BEA . 1990 Two-photon interference in a Mach-Zehnder interferometer. Phys. Rev. Lett. **65** , 1348–1351. (10.1103/PhysRevLett.65.1348) 10042241

[12] Franson J . 1989 Bell inequality for position and time. Phys. Rev. Lett. **62** , 2205–2208. (10.1103/PhysRevLett.62.2205) 10039885

[13] Wang LJ , Zou XY , Mandel L . 1991 Induced coherence without induced emission. Phys. Rev. A **44** , 4614–4622. (10.1103/physreva.44.4614) 9906504

[14] Herzog TJ , Rarity JG , Weinfurter H , Zeilinger A . 1994 Frustrated two-photon creation via interference. Phys. Rev. Lett. **72** , 629–632. (10.1103/PhysRevLett.72.629) 10056483

[15] Rarity JG . 1995 Interference of single photons from separate sources. Ann. N. Y. Acad. Sci. **755** , 624–631. (10.1111/j.1749-6632.1995.tb39002.x)

[16] Zukowski M , Zeilinger A , Weinfurter H . 1995 Entangling photons radiated by independent pulsed sourcesa. Ann. NY Acad. Sci. **755** , 91–102. (10.1111/j.1749-6632.1995.tb38959.x)

[17] Rarity JG , Tapster PR . 1996 Interference between a single photon and a superposition of coherent states. In Quantum interferometry (eds Fd Martini , G Denardo , Y Shih, ), p. 211, vol. **355** . Weinheim: Wiley VCH.

[18] Rarity JG , Tapster PR , Loudon R . 2005 Non-classical interference between independent sources. J. Opt. B Quantum Semiclass. Opt. **7** , S171–S175. (10.1088/1464-4266/7/7/007)

[19] Bouwmeester D , Pan JW , Mattle K , Eibl M , Weinfurter H , Zeilinger A . 1997 Experimental quantum teleportation. Nature **390** , 575–579. (10.1038/37539)

[20] Pan JW , Bouwmeester D , Weinfurter H , Zeilinger A . 1998 Experimental entanglement swapping: entangling photons that never interacted. Phys. Rev. Lett. **80** , 3891–3894. (10.1103/PhysRevLett.80.3891)

[21] Pan JW , Chen ZB , Lu CY , Weinfurter H , Zeilinger A , Żukowski M . 2012 Multiphoton entanglement and interferometry. Rev. Mod. Phys. **84** , 777–838. (10.1103/RevModPhys.84.777)

[22] Ou ZY . 2007 Multi-photon quantum interference. New York, NY: Springer. (10.1007/978-0-387-25554-5)

[23] Loudon R . 1973 Quantum theory of light. Oxford, UK: Oxford University Press.

[24] Glauber RJ . 1963 The quantum theory of optical coherence. Phys. Rev. **130** , 2529–2539. (10.1103/PhysRev.130.2529)

[25] Rarity JG , Tapster PR . 1989 Fourth-order interference in parametric downconversion. J. Opt. Soc. Am. B **6** , 1221. (10.1364/JOSAB.6.001221)

[26] Clauser JF , Horne MA , Shimony A , Holt RA . 1969 Proposed experiment to test local hidden-variable theories. Phys. Rev. Lett. **23** , 880–884. (10.1103/PhysRevLett.23.880)

[27] Weinfurter H . 1994 Experimental bell-state analysis. Europhys. Lett. **25** , 559–564. (10.1209/0295-5075/25/8/001)

[28] Knill E , Laflamme R , Milburn GJ . 2001 A scheme for efficient quantum computation with linear optics. Nature **409** , 46–52. (10.1038/35051009) 11343107

[29] O’Brien JL , Pryde GJ , White AG , Ralph TC , Branning D . 2003 Demonstration of an all-optical quantum controlled-NOT gate. Nature **426** , 264–267. (10.1038/nature02054) 14628045

[30] Sharping JE , Lee KF , Foster MA , Turner AC , Schmidt BS , Lipson M , Gaeta AL , Kumar P . 2006 Generation of correlated photons in nanoscale silicon waveguides. Opt. Express **14** , 12388–12393. (10.1364/oe.14.012388) 19529670

[31] Pittman TB , Jacobs BC , Franson JD . 2001 Probabilistic quantum logic operations using polarizing beam splitters. Phys. Rev. A **64** , 062311. (10.1103/PhysRevA.64.062311)

[32] Browne DE , Rudolph T . 2005 Resource-efficient linear optical quantum computation. Phys. Rev. Lett. **95** , 010501. (10.1103/PhysRevLett.95.010501) 16090595

[33] Adcock JC , Vigliar C , Santagati R , Silverstone JW , Thompson MG . 2019 Programmable four-photon graph states on a silicon chip. Nat. Commun. **10** , 3528. (10.1038/s41467-019-11489-y) 31388017 PMC6684799

[34] Llewellyn D *et al* . 2019 Chip-to-chip quantum teleportation and multi-photon entanglement in silicon. Nat. Phys. **16** , 148–153. (doi:10.1038/s41567-019- 0727-x)

[35] Vigliar C *et al* . 2021 Error-protected qubits in a silicon photonic chip. Nat. Phys. **17** , 1137–1143. (10.1038/s41567-021-01333-w)

[36] Wang J , Sciarrino F , Laing A , Thompson MG . 2020 Integrated photonic quantum technologies. Nat. Photonics **14** , 273–284. (10.1038/s41566-019-0532-1)

[37] Raussendorf R , Briegel HJ . 2001 A one-way quantum computer. Phys. Rev. Lett. **86** , 5188–5191. (10.1103/PhysRevLett.86.5188) 11384453

[38] Gimeno-Segovia M , Shadbolt P , Browne DE , Rudolph T . 2015 From three-photon greenberger-horne-zeilinger states to ballistic universal quantum computation. Phys. Rev. Lett. **115** , 020502. (10.1103/PhysRevLett.115.020502) 26207455

[39] Rudolph T . 2017 Why I am optimistic about linear optics quantum computing. APL Photon. **2** , 030901. (10.1063/1.4976737)

[40] Grice WP , U’Ren AB , Walmsley IA . 2001 Eliminating frequency and space-time correlations in multiphoton states. Phys. Rev. A **64** , 063815. (10.1103/PhysRevA.64.063815)

[41] Paesani S , Borghi M , Signorini S , Maïnos A , Pavesi L , Laing A . 2020 Near-ideal spontaneous photon sources in silicon quantum photonics. Nat. Commun. **11** , 2505. (10.1038/s41467-020-16187-8) 32427911 PMC7237445

[42] Burridge BM , Faruque II , Rarity JG , Barreto J . 2023 Integrate and scale: a source of spectrally separable photon pairs. Optica **10** , 1471. (10.1364/OPTICA.491965)

[43] Alexander K *et al* . 2024 A manufacturable platform for photonic quantum computing. arXiv https://arxiv.org/pdf/2404.17570 10.1038/s41586-025-08820-7 PMC12095036 40010377

[44] Dirac PAM . 1930 The principles of quantum mechanics, p. 9, 4th edn. Oxford, UK: Clarendon.

[45] Pfleegor RL , Mandel L . 1967 Interference of independent photon beams. Phys. Rev. **159** , 1084–1088. (10.1103/PhysRev.159.1084)

[46] Glauber RJ . 1995 Dirac’s famous dictum on interference: one photon or two? Am. J. Phys. **63** , 12. (10.1119/1.17790)

[47] Shor PW . 1994 Algorithms for quantum computation: discrete logarithms and factoring. In Proc. 35th Annual Symposium on Foundations of Computer Science, pp. 124–134. Santa Fe, NM: IEEE. (10.1109/SFCS.1994.365700)

